# EUCAST Ibrexafungerp MICs and Wild-Type Upper Limits for Contemporary Danish Yeast Isolates

**DOI:** 10.3390/jof8101106

**Published:** 2022-10-20

**Authors:** Karin M. Jørgensen, Karen M. T. Astvad, Rasmus K. Hare, Maiken C. Arendrup

**Affiliations:** 1 Unit of Mycology, Statens Serum Institut, DK-2300 Copenhagen, Denmark; 2 Department of Clinical Microbiology, Rigshospitalet, DK-2100 Copenhagen, Denmark; 3 Department of Clinical Medicine, University of Copenhagen, DK-2100 Copenhagen, Denmark

**Keywords:** *Candida*, echinocandins, ECOFF, WT-UL

## Abstract

Ibrexafungerp is a novel triterpenoid antifungal that inhibits glucan synthase and thus fungal cell wall synthesis. We examined the in vitro activity against contemporary clinical yeast, investigated inter-laboratory and intra-laboratory variability, suggested wild-type upper-limit values (WT-UL), and compared in vitro activity of ibrexafungerp to five licensed antifungals. Susceptibility to ibrexafungerp and comparators was investigated prospectively for 1965 isolates (11,790 MICs) and repetitively for three QC strains (1764 MICs) following the EUCAST E.Def 7.3.2 method. Elevated ibrexafungerp/echinocandin MICs prompted *FKS* sequencing. Published ibrexafungerp EUCAST MIC-distributions were retrieved and aggregated for WT-UL determinations following EUCAST principles. Ibrexafungerp MICs were ≤2 mg/L except against *C. pararugosa, Cryptococcus* and some rare yeasts. Modal MICs (mg/L) were 0.06/0.125/0.25/0.5/0.5/0.5/0.5/1/2 for *C. albicans*/*C. dubliniensis*/*C. glabrata*/*C. krusei*/*C. parapsilosis*/*C. tropicalis*/*S. cerevisiae*/*C. guilliermondii*/*C. lusitaniae* and aligned within ±1 dilution with published values. The MIC ranges for QC strains were: 0.06–0.25/0.5–1/0.125–0.5 for CNM-CL-F8555/ATCC6258/ATCC22019. The WT-UL (mg/L) were: 0.25/0.5/1/1/2 for *C. albicans*/*C. glabrata*/*C. krusei*/*C. parapsilosis*/*C. tropicalis*. Adopting these, non-wild-type rates were 0.3%/0.6%/0%/8%/3% for *C. albicans*/*C. glabrata*/*C. krusei*/*C. parapsilosis*/*C. tropicalis* and overall lower than for comparators except amphotericin B. Five/six non-wild-type *C. albicans*/*C. glabrata* were echinocandin and Fks non-wild-type (F641S, F659del or F659L). Eight *C. parapsilosis* and three *C. tropicalis* non-wild-type isolates were echinocandin and Fks wild-type. Partial inhibition near 50% in the supra-MIC range may explain variable MICs. Ibrexafungerp EUCAST MIC testing is robust, although the significance of paradoxical growth for some species requires further investigation. The spectrum is broad and will provide an oral option for the growing population with azole refractory infection.

## 1. Introduction

Ibrexafungerp (formerly SCY-078) is a novel triterpenoid antifungal that interferes with the fungal cell wall synthesis through inhibition of glucan synthase [1]. It has fungicidal activity against *Candida*, including the often multidrug-resistant species *Candida auris* and fungistatic activity against *Aspergillus* [2,3,4,5]. Moreover, ibrexafungerp has activity against the ascus form of *Pneumocystis jirovecii* and has displayed activity in a murine prophylaxis model of pneumocystosis [6]. Ibrexafungerp is available in oral formulations and was approved in 2021 by the FDA for treatment of *Candida* vaginitis. A liposomal intravenous formulation is under development and ibrexafungerp is currently in clinical studies for treatment of refractory or intolerant fungal diseases, for *C. auris* infections, for treatment of invasive pulmonary aspergillosis in combination therapy with voriconazole, and for recurrent vulvovaginal candidiasis. The mode of action is comparable to that of the echinocandins. The binding site is different from, but overlapping with that of the echinocandins leading to cross-resistance to some but not all *FKS* mutations in *Candida* spp. [3,7].

Clinical breakpoints have not yet been set for ibrexafungerp against *Candida* spp. An obligatory ingredient in breakpoint setting is analysis of MIC distributions from multiple sources and determination of epidemiological cut off values (ECOFF in EUCAST terminology and ECV in CLSI terminology). These are defined as the highest MIC value for isolates devoid of phenotypically detectable acquired resistance mechanisms, also called wild-type isolates [8]. The ECOFFs do not inform on clinical susceptibility because they only reflect the inherent susceptibility of the species, but they inform on the likelihood of presence of acquired resistance mechanisms that may or may not affect outcome depending on the drug exposure during therapy. Microbroth susceptibility testing is associated with technical variation related to differences between products from different manufacturers (such as brand of microtitre plates, medium and characteristics of the antifungal agent), method used for serial dilution, differences in how materials are made or handled, differences between how different individuals perform the same test, differences in cell density in the inoculum, differences in temperature stability of incubators, differences in atmospheres in incubators, etc. [8,9,10,11]. To encompass this variation, EUCAST requires at least 100 MICs from at least five independent sources, each consisting of at least 15 isolates and for which the modal MIC may not deviate more than one two-fold dilution from the most common mode for dataset used for EUCAST ECOFF setting [8]. So far, three studies have reported single or multicentre MICs [12,13,14]. The objectives of this study were to examine the in vitro activity of ibrexafungerp against a nationwide and representative sample of contemporary clinical yeast isolates of *Candida*, to compare the MICs obtained with those recently reported and set preliminary wild-type upper limits (WT-UL), and to compare the activity of ibrexafungerp to that of other agents licensed for the therapy of candidiasis.

## 2. Materials and Methods

**Isolates.** All unique yeast isolates received at the Statens Serum Institut (SSI) as pure cultures or cultured from clinical samples during 2020 and 2021 were included. Same patient same species isolates were regarded as unique if obtained >21 days apart or with a different susceptibility pattern. 

**Susceptibility testing and target gene sequencing.** EUCAST E.Def 7.3.2 MIC determination was performed for ibrexafungerp and five comparators with *Candida krusei* ATCC 6258, *Candida parapsilosis* ATCC 22019 and *Candida albicans* CNM CL-F8555 as QC strains [15]. The MIC determination was performed prospectively during 2020–2021 using multiple batches of in-house prepared trays. Cell culture treated (Nunc™ MicroWell™ 96-Well Microplates, ThermoFisher Scientific cat. no. 167008/161093) were used throughout. Microtitre plates with 2-fold dilutions were prepared using serial dilution and two pipette tip changes and frozen at −80 °C for at least 24 h prior to use [10]. Ibrexafungerp (SCY-078, Scynexis Inc., Jersey City, NJ, USA) pure substance was stored in aliquots at −80 °C and stock solutions prepared in DMSO (Sigma-Aldrich, Brøndby, Denmark, 5000 mg/L). The final drug concentration range studied was 0.008–8 mg/L. The following comparator compounds were also investigated (source of compound and final concentration range in parentheses). Anidulafungin (Pfizer A/S, Ballerup, Denmark, 0.004–4 mg/L), micafungin (Astellas Pharma Inc., Tokyo, Japan until August 2021, then from Molcan corporation, Toronto, Canada, 0.004–4 mg/L), amphotericin B (Sigma-Aldrich, 0.004–4 mg/L), fluconazole (Sigma-Aldrich, either 0.06–64 mg/L), and voriconazole (Pfizer A/S, Ballerup, Denmark, 0.004–4 mg/L). The following quality control (QC) strains (number of repetitions) were included: *C. albicans* CNM-CL-F8555 (n = 79), *C. krusei* ATCC 6258 (n = 116) and *C. parapsilosis* ATCC 22019 (n = 99) as quality controls for the comparators and to generate QC MIC data for Ibrexafungerp (1764 MICs for QC strains in total).

*FKS* sequencing was performed for echinocandin-resistant isolates as previously described [16].

**Data management.** The MIC ranges, modal MIC (the most common MIC), and MIC_50_ and MIC_90_ (the MIC value that includes 50% and 90% of the isolates, respectively) values were determined for ibrexafungerp and comparators (anidulafungin, micafungin, amphotericin B, fluconazole and voriconazole). Published EUCAST MIC distributions were retrieved and inspected for agreement with data from this study according to the EUCAST SOP 10.2 [8]. Ibrexafungerp WT-UL values, defined as the upper MIC value where the wild-type distribution ends, were determined visually and statistically using the EUCAST ECOFFinder programme and inclusion of 97.5% to 99.9% of the predicted wild-type population for species-specific MIC distributions with ≥15 isolates [17]. However, as the values reported here are not formally accepted EUCAST ibrexafungerp ECOFFs, we used the term “WT-UL” to avoid confusion. 

## 3. Results

**Ibrexafungerp activity against clinical isolates.** In total, 1965 yeast isolates (hereof 1893 *Candida* spp. isolates) were included and tested prospectively in parallel with repetitive testing of three quality control strains during 2020 and 2021 using multiple batches of in-house-prepared microdilution EUCAST plates. The number of clinical isolates per year was comparable with 967 isolates, including 540 from blood cultures, from 794 patients in 2020, and 1001 isolates, including 561 blood isolates, from 824 patients in 2021, respectively. The modal MICs were identical comparing MIC distributions from 2020 and 2021 for all species represented with at least 10 isolates (*C. albicans*, *C. dubliniensis*, *C. glabrata*, *C. krusei*, *C. parapsilosis*, *C. tropicalis*, *C. guilliermondii*, *C. lusitaniae* and *S. cerevisiae*) and hence the data from the two years were compiled (Table 1). As expected for Gaussian distributions, the modal MIC and MIC_50_ values were identical for each species: *C. albicans*: 0.06 mg/L, *C. dubliniensis* 0.125 mg/L, *C. glabrata* 0.25 mg/L and for *C. krusei*, *C. parapsilosis, C. tropicalis* and *S. cerevisiae* all 0.5 mg/L. *C. guilliermondii, C. lusitaniae* and *C. pararugosa* were less susceptible (modal MIC/MIC_50_ values of 1, 2 and 8 mg/L, respectively). Among the rare yeast, ibrexafungerp MICs ≤ 2 mg/L were found for *Arxula adeninivorans* (n = 1), *Lodderomyces elongisporus* (n = 1), *Pichia manshurica* (n = 1), *Wickerhamomyces anomalus* (*Candida pelliculosa*) (n = 2), *Wickerhamomyces onychis* (n = 1), *Yarrowia lipolytica* (*Candida lusitaniae*)(n = 1), *Kodamaea ohmeri* (n = 1) and *Wickerhamomyces* species (n = 1) whereas the majority of the MICs against the remaining rare yeast species were ≥4 mg/L (Supplementary Table S1).

**Activity against quality control strains.** The MIC ranges for ibrexafungerp against all three QC strains spanned two to three two-fold dilutions both years (Table 2). The ibrexafungerp modal MICs (mg/L) in 2020, 2021 and combined were: *C. albicans* CNM-CL F8555: 0.125; 0.125 and 0.125, *C. krusei* ATCC 6258: 0.5; 1 and 0.5 and *C. parapsilosis* ATCC 22019: 0.25; 0.25 and 0.25. In comparison, the MIC ranges for the comparators spanned two to four two-fold dilutions for amphotericin B, three to four dilutions for anidulafungin, two to four dilutions for micafungin, three dilutions for fluconazole and three to four dilutions for voriconazole. At least 95% of the MICs for comparators fell within the established MIC ranges for the three QC strains for amphotericin B, anidulafungin and voriconazole but fewer 79% for micafungin against *C. krusei* ATCC 6258 in 2020 and 31–46% for fluconazole against *C. albicans* CNM-CL F8555 in 2020 and 2021, specifically.

**Inter-laboratory agreement for ibrexafungerp MIC testing**. Next, the ibrexafungerp MIC distributions were compared to those from three recent publications (Table 3) [12,13,14]. All MIC distributions were unimodal with the exception of a bimodal MIC distribution for *C. albicans* (modes 0.03 and 0.125 mg/L) reported in the multicentre study by Quindos et al. [14], and which therefore was excluded from the WT-UL determination for the aggregated data. The species-specific modal MICs all fell within ± 1 two-fold dilution from the most common species-specific modal MIC. The modal MICs for the aggregated dataset were for all species identical to the ones obtained in this study: *C. albicans*: 0.06 mg/L, *C. glabrata:* 0.25 mg/L, *C. krusei*: 0.5 mg/L, *C. parapsilosis*: 0.5 mg/L and *C. tropicalis* 0.5 mg/L.

**WT-UL determination and wild-type non-wild-type classification.** WT-ULs were determined statistically including 97.5%, 99%, 99.5% and 99.9% of the modelled aggregated wild-type distributions, and visually. The best agreement between the statistical and visual values was found for the WT-UL including 99.5%, which was chosen for wild-type versus non-wild-type classification. A single-centre WT-UL was also determined for *C. dubliniensis* MICs from this study following the same criteria (Table 4). 

Applying the consensus WT-UL to the Danish isolates, three *C. albicans*, one *C. dubliniensis*, three *C. glabrata*, eight *C. parapsilosis* and three *C. tropicalis* were classified as non-wild-type isolates (Table 1 and Table 3). The MIC and Fks information for these isolates are detailed in Table 5, together with isolates that were found wild-type for ibrexafungerp but anidulafungin- and micafungin-resistant. Among *C. albicans* isolates with ibrexafungerp MIC 1–2 mg/L, two of three were resistant to anidulafungin and micafungin, and both harboured an F641S alteration in Fks1. The third isolate was anidulafungin, micafungin and *FKS* wild-type. Among *C. albicans* isolates with ibrexafungerp MIC 0.25 mg/L and wild-type to ibrexafungerp, two of ten were resistant to anidulafungin and micafungin. Both isolates were *FKS*-sequenced and harboured alterations known to confer echinocandin resistance (S645P and R1361H, respectively), whereas the remaining eight were anidulafungin and micafungin wild-type and not *FKS*-sequenced. For *C. glabrata* with ibrexafungerp MICs 1-2 mg/L, three of three isolates were resistant to anidulafungin or micafungin, and all three harboured alterations involving F659 in Fks2. Eleven *C. glabrata* isolates were ibrexafungerp wild-type (MIC 0.25–0.5 mg/L) but were resistant to anidulafungin or micafungin. Among these, two harboured an F659Y alteration (ibrexafungerp MIC 0.25 and 0.5 mg/L, respectively), eight isolates harboured alterations involving codons further downstream in Fks1 or Fks2 hot spot regions (Table 5) and one isolate had alteration (K1323E)17 amino acids prior to Fks1 hot spot two. For the remaining species with MICs above the WT-ULs, one of one *C. dubliniensis*, eight of eight *C. parapsilosis* and three of three *C. tropicalis* isolates were anidulafungin- and micafungin- susceptible. Seven of these were *FKS*-sequenced and confirmed without mutations in the echinocandin hotspots or regions upstream thereof (Table 5). 

Two additional isolates were found non-wild-type to echinocandins. One was a *C. krusei* wild-type for ibrexafungerp but with a micafungin MIC of 2 mg/L and an S659P alteration. The other was a *C. auris* isolate with anidulafungin and micafungin resistance and an Fks1 F635Y alteration [18]. The ibrexafungerp MIC of this isolate was 1 mg/L, and thus only one two-fold dilution higher than the modal MIC determined in our study of ibrexafungerp EUCAST susceptibility of *C. auris* [3].

**Growth patterns for ibrexafungerp non-wild-type but echinocandin-susceptible isolates.** A total of 13 isolates (one *C. albicans*, one *C. dubliniensis*, eight *C. parapsilosis* and three *C. tropicalis*) were deemed ibrexafungerp non-wild-type but anidulafungin- and micafungin-susceptible and *FKS* wild-type if sequenced. The growth curves of these isolates were inspected and compared to those for selected wild-type isolates (Supplementary Figure S1a–h). The growth curves for repeated testing of the *C. albicans* isolate were characterised by complete inhibition in the presence of 8 and 4 mg/L ibrexafungerp, by partial inhibition close to 50% in the 2–0.25 mg/L concentration range, and by decreasing inhibition with decreasing concentrations thereafter (Figure S1a). This led to inconsistent MIC determination in three runs: 0.25 mg/L, 2 mg/L and 4 mg/L, respectively, depending on where the curve intersected the 50% endpoint in the range of partial inhibition. For comparison, the growth curves for five *C. albicans* isolates without partial inhibition but with differential susceptibility are shown in Figure S1b. The *C. dubliniensis* isolate displayed a steadily rising growth curve with decreasing concentrations on repeat testing (Figure S1c). Three randomly selected *C. dubliniensis* with MIC 0.125 mg/L are shown in Figure S1d and show a characteristic paradoxical growth in supra MIC concentrations 1–4 mg/L but that does not exceed the 50% cut-off line. Growth curves for the eight *C. parapsilosis* isolates with MICs 4–8 mg/L when applying a 50% endpoint are shown in Figure S1e and compared to repetitive testing of the *C. parapsilosis* QC strain ATCC 22019 in Figure S1f. Complete inhibition is seen in the presence of 8 mg/L, partial inhibition in the 0.5–4 mg/L range and loss of activity in the following decreasing concentrations. The level of growth in the partial inhibition area was variable on repeated testing and again led to random MICs depending on if the level exceeded the 50% cut-off line or not. Finally, the three *C. tropicalis* isolates were compared to three selected isolates where the growth curve intersected the 50% growth line several times complicating endpoint reading, and three randomly chosen isolates displaying paradoxical growth at supra MIC concentrations but not above the 50% (Figure S1g,h).

**Susceptibility to comparators.** Amphotericin B was the agent with the broadest activity, as resistance was only found against four *Trichosporon* spp. isolates out of the thirty-four rare yeast (Table 6). Fluconazole resistance was found among all *Candida* species except *C. kefyr* and *C. lusitaniae* and with acquired resistance ranging from 2.6% in *C. albicans*, *C. dubliniensis* and *C. tropicalis* to over 6.4% in *C. parapsilosis* to 10.1% in *C. glabrata*. A notable number of *Candida* isolates were resistant to voriconazole with rates varying from 0.9% in *C. dubliniensis* to 6.4 and 6.6% in *C. parapsilosis* and *C. glabrata*, respectively. Finally, acquired resistance to anidulafungin/micafungin was found in *C. albicans* 1.0/1.8%, *C. glabrata* 2.3/3.6%, *C. krusei* 0.9/0.9% and *C. parapsilosis* 0/2.6%.

## 4. Discussion

The main findings in this study were (1) that ibrexafungerp susceptibility testing during 2 years of routine testing conditions was robust, and that inter-laboratory agreement was high when compared to published data, (2) that WT-ULs could be set and adopted for classification into wild-type and non-wild-type phenotypes until formal EUCAST ECOFFs and clinical breakpoints have been established, (3) that ibrexafungerp displays broad activity against all the included *Candida* spp. except *C. pararugosa*, (4) that ibrexafungerp retains activity against most *FKS* mutant isolates found in our laboratory during 2020–2021, and (5) that only amphotericin B displayed a lower resistance rate in agreement with the almost universal activity of this agent. However, the study also identified some technical challenges that may lead to random classification of isolates, particularly for *C. dubliniensis*, *C. parapsilosis* and *C. tropicalis* due to partial growth inhibition in a supra-MIC range for EUCAST testing.

The ibrexafungerp MIC testing was robust as documented by narrow and comparable MIC distributions for QC strains and identical modal MICs for clinical isolates across the two years. This suggests a stable and robust intra-laboratory performance of the EUCAST method. The modal MICs obtained in this study were identical to or one dilution from the modal MIC both for the two QC strains also repeatedly tested by Mesquida et al. [12], and for the common *Candida* species included in recent single and multicentre studies of ibrexafungerp EUCAST susceptibility [12,13,14]. These observations suggest a good inter-laboratory agreement. Of note however, a bimodal MIC distribution was noted in the multicentre study for *C. albicans* specifically, with a first peak at 0.03 mg/L and another at 0.125 mg/L suggesting that some optimisation may be warranted, which will be facilitated once QC MIC target and ranges have been established [14].

The activity of ibrexafungerp was broad and more uniform than for echinocandins against *Candida* spp. with *C. pararugosa* being the only clearly ibrexafungerp-resistant *Candida* species in our study. The activity included the rarer species *S. cerevisiae, A. adeninivorans*, *L*. *elongisporus*, *P. manshurica*, *W. anomalus* (*C. pelliculosa*), *W. onychis* and *Y. lipolytica* (*C*. *lipolytica*), which were susceptible at concentrations up to 2 mg/L. The inherent echinocandin susceptibility of *C. parapsilosis* is notably different compared to that for other *Candida* species (e.g., eight two-fold dilutions for anidulafungin MICs in this study).

In contrast, the ibrexafungerp MICs are more discretely elevated for *C. parapsilosis* compared to *C. albicans* (three dilutions) and comparable to those against *C. tropicalis* and *C. krusei*. *C. parapsilosis* harbours an intrinsic amino acid substitution, alanine A660, at the last position of the hot spot region in Fks1 in contrast to proline in the other *Candida* species (P649 in *C. albicans*). This aligns with the fact that the binding site for ibrexafungerp is earlier compared to that for echinocandins and hence not affected by alterations in the mid and last parts of the hotspot region [19].

Adopting the WT-UL values based upon the aggregated data from this study and three others [12,13,14], we found 18 isolates among the common species with non-wild-type MIC. Five of six were *C. albicans* or *C. glabrata* with alterations at the first codon in hot spot one of Fks 1 or Fks2 (*C. albicans* F641S (n = 2), *C. glabrata* F659del (n = 2) and F659L (n = 1)), previously associated with ibrexafungerp echinocandin cross-resistance [7,20]. However, we also found two *C. glabrata* and one *C. auris* with F659Y and F635Y alterations, respectively, which were echinocandin-resistant but ibrexafungerp wild-type. Pfaller et al. found elevated ibrexafungerp MICs for *C. albicans* isolates harbouring F641I, F641Y and F641S in Fks1, *C. glabrata* harbouring F625S, but not F625Y, in Fks1, and F659V in Fks2 and in 2/3 *C. tropicalis* isolates harbouring the F641S alteration in Fks1 [13]. Mesquida et al. found three isolates with non-wild-type MICs of which the *C. glabrata* isolates had a F659S alteration but the *C. albicans* and *C. tropicalis* isolates did not harbour Fks alterations among their blood stream isolates [12,20]. These data support that alterations at the phenylalanine (F) codon can cause echinocandin and/or ibrexafungerp resistance but not universally and that some resistant isolates do not harbour Fks alterations. Obviously, this will complicate a translation of molecular data to phenotypic susceptibility pattern. None of our 12 *C. dubliniensis*, *C. parapsilosis* or *C. tropicalis* isolates with elevated ibrexafungerp MICs harboured Fks alterations or displayed resistance to the echinocandins. For a number of these, we believe that a paradoxical growth in the 0.5 to 4 mg/L range led to a random wild-type versus non-wild-type classification depending on if the growth curve intersected the 50% inhibition line or not. Of note, this was not observed for the highest concentration tested (8 mg/L), which is somewhat in contrast to the paradoxical effect described for echinocandins [21]. We also hypothesise that this is not only an issue in our laboratory, as wide MIC distributions for *C. parapsilosis* and *C. tropicalis* were also reported by Quindos et al. [14]. Obviously, further studies are warranted to explore whether this is solely a technical issue or has potential clinical aspects.

As expected for a new drug class, non-wild-type isolates were less common than for echinocandins and azoles. This is indeed promising, with currently no oral alternatives for azole-resistant infections, and at a time where fluconazole resistance is emerging in both *C. glabrata* and *C. parapsilosis* [22,23,24,25].

## 5. Conclusions

Ibrexafungerp displayed broad activity against Danish isolates including most *FKS* mutant *Candida* isolates and species with inherent or acquired resistance to fluconazole. This is a promising prospect for many patients for whom we today have no oral options. WT-ULs were set and MICs for QC strains presented, which will allow for comparison and interpretation until formal values have been set by EUCAST. A partial inhibition pattern was observed against some isolates, particularly those of *C. parapsilosis* but also *C. dubliniensis* and *C. tropicalis*, which complicated MIC determination. Whether this reflects different clinical susceptibility or a technical issue related to in vitro testing warrants further investigation. 

## Figures and Tables

**Table 1:** Ibrexafungerp MICs (mg/L) for 1965 yeast isolates (incl. 1893 *Candida* spp. isolates) and three other species isolates from Denmark collected in 2020 and 2021 combined.

Species	N	MIC (mg/L)											MIC_50_	MIC_90_	MIC Range	% NWT
		0.016	0.03	0.06	0.125	0.25	0.5	1	2	4	8	>8				
*Candida*																
*C. albicans*	896	5	91	574	213	10		**2 ^**	**1**				0.06	0.125	0.016–2	0.3
*C. dubliniensis*	117			15	64	33	4		**1**				0.125	0.25	0.06–2	0.9
*C. glabrata*	475				5	319	148	**2 ^**	**1 ^**				0.25	0.5	0.125–2	0.6
*C. krusei*	110					14	74	22					0.5	1	0.25–1	0
*C. parapsilosis*	78				1	23	44	2	**1**	**6**	**1**		0.5	2	0.125–8	10.3
*C. tropicalis*	146				8	44	75	16		**2**	**1**		0.5	1	0.125–8	2.1
*C. guilliermondii*	12						1	10	1				1	NA	0.5–2	NA
*C. kefyr*	8				1	5	2						0.25	NA	0.125–0.5	NA
*C. lusitaniae*	19							7	11	1			2	2	1–4	0
Other *Candida* #	33				2	16	4	10			1		NA	NA	0.125–8	NA
Other yeast																
*S. cerevisiae*	30				1	10	19						0.5	0.5	0.125–0.5	NA
*Cryptococcus* spp.	7								5	2			2	NA	2–4	NA
Rare yeast *	34		1			3	3	3	2	3	13	6	NA	NA	0.03–>8	NA
In total	1965	5	92	589	295	477	374	74	23	14	16	6				NA

Modal MICs are underlined; non-wild-type (NWT) MIC defined as MICs above the consensus WT-UL (see WT-UL determination section below) are highlighted using bold font. ^ indicates ibrexafungerp non-wild-type isolates that harbour Fks amino acid (AA) alterations: *C. albicans*: both isolates F641S, *C. glabrata*: two isolates with F659del and one with F659L. # Other *Candida* included the following species with ibrexafungerp MIC in the 0.125–0.5 range: *C. metapsilosis* (one), *C. orthopsilosis* (eight), *C. lambica* (one), *C. norvegensis* (six), *C. pelliculosa* (two), *C. utilis* (one); the following species with ibrexafungerp MIC in the 0.5–2 range: *C. intermedia* (one), *C. stellimalicola* (one) *C. auris* (two), *C. fermentati* (four), *C. inconspicua* (one), *C. lipolytica* (one), *C. melibiosica* (one), *C. pulcherrima* (one), *C. sorbosivorans* (one); and finally one *C. pararugosa* with an MIC of 8 mg/L. * Rare yeast included the following species with ibrexafungerp MIC ≤ 0.5 range: *Arxula adeninivorans* (one), *Lodderomyces elongisporus* (one), *Pichia manshurica* (one), *Wickerhamomyces anomalus* (two), *Wickerhamomyces onychis* (one) and *Yarrowia lipolytica* (one); the following species with ibrexafungerp MIC in the 0.5–2 range: *Wickerhamomyces* species (one); and finally the following species that included isolates with the majority of MICs being 4 mg/L or greater: *Magnusiomyces capitatus* (five), *Trichosporon asahii* (four), *Geotrichum candidum* (five), *Rhodotorula mucilaginosa* (three), *Exophiala dermatitidis* (one), *Lachancea thermotolerans* (one), *Trichosporon dermatis* (two), *Geotrichum capitatum* (two), *Geotrichum species* (two) and *Geotrichum silvicola* (one). NA: Not applicable, MIC_50_ values were determined for all species-specific MIC distributions. MIC_90_ values for species with ≥15 isolates.

**Table 2:** MIC values for ibrexafungerp and comparators against three QC strains that were tested repetitively throughout the 2-year study period in parallel with the clinical isolates.

Compound	MIC (mg/L)															% within Range
QC Strain, Year (N. of Repetitions)	≤0.004	0.008	0.016	0.03	0.06	0.125	0.25	0.5	1	2	4	8	16	32	64	
**Ibrexafungerp**																
*C. albicans* CNM-CL F8555, 2020 (37)					16	**20**	1									
*C. albicans* CNM-CL F8555, 2021 (42)					16	**25**	1									
*C. krusei* ATCC 6258, 2020 (68)								**43**	25							
*C. krusei* ATCC 6258, 2021 (48)								23	**25**							
*C. parapsilosis* ATCC 22019, 2020 (55)						4	**32**	19								
*C. parapsilosis* ATCC 22019, 2021 (44)							**31**	13								
**Amphotericin B**																
*C. albicans* CNM-CL F8555, 2020 (37)						10	**23**	4								100%
*C. albicans* CNM-CL F8555, 2021 (42)						12	**30**									100%
*C. krusei* ATCC 6258, 2020 (68)							0	**36**	32							100%
*C. krusei* ATCC 6258, 2021 (48)							5	**38**	5							100%
*C. parapsilosis* ATCC 22019, 2020 (55)						3	16	**33**	3							100%
*C. parapsilosis* ATCC 22019, 2021 (44)						1	**23**	19	1							100%
**Anidulafungin**																
*C. albicans* CNM-CL F8555, 2020 (37)	**21**	16														
*C. albicans* CNM-CL F8555, 2021 (42)	**37**	5														
*C. krusei* ATCC 6258, 2020 (68)			6	**57**	5											100%
*C. krusei* ATCC 6258, 2021 (48)			9	**39**												100%
*C. parapsilosis* ATCC 22019, 2020 (55)						1	5	**42**	7							98%
*C. parapsilosis* ATCC 22019, 2021 (44)							7	**35**	2							100%
**Micafungin**																
*C. albicans* CNM-CL F8555, 2020 (37)			15	**22**												
*C. albicans* CNM-CL F8555, 2021 (42)			**31**	11												
*C. krusei* ATCC 6258, 2020 (68)					0	**54**	14									79%
*C. krusei* ATCC 6258, 2021 (48)					0	**47**	1									98%
*C. parapsilosis* ATCC 22019, 2020 (55)							1	6	**43**	5						98%
*C. parapsilosis* ATCC 22019, 2021 (44)								2	**36**	6						100%
**Fluconazole**																
*C. albicans* CNM-CL F8555, 2020 (37)													**20**	17	0	46%
*C. albicans* CNM-CL F8555, 2021 (42)													**29**	12	1	31%
*C. krusei* ATCC 6258, 2020 (68)													5	**59**	4	100%
*C. krusei* ATCC 6258, 2021 (48)													1	**42**	5	100%
*C. parapsilosis* ATCC 22019, 2020 (55)									14	**38**	3					95%
*C. parapsilosis* ATCC 22019, 2021 (44)									14	**28**	2					95%
**Voriconazole**																
*C. albicans* CNM-CL F8555, 2020 (37)							2	**33**	2							95%
*C. albicans* CNM-CL F8555, 2021 (42)							4	**36**	1	1						90%
*C. krusei* ATCC 6258, 2020 (68)					1	10	**57**									100%
*C. krusei* ATCC 6258, 2021 (48)					0	4	**42**	2								96%
*C. parapsilosis* ATCC 22019, 2020 (55)			8	**43**	4											100%
*C. parapsilosis* ATCC 22019, 2021 (44)			1	**29**	14											100%

The concentration range tested varied by agent. Grey colour indicates concentrations not tested. The modal MICs for the MICs obtained in this study are highlighted in bold font. The recommended EUCAST MIC range and target MIC for the QC strains are indicated in green shading and underlined font, respectively, as summarised in “The European Committee on Antimicrobial Susceptibility Testing. Routine and extended internal quality control for MIC determination and agar dilution for yeasts, moulds and dermatophytes as recommended by EUCAST. Version 6.0, 2022. http://www.eucast.org”.

**Table 3:** Ibrexafungerp MICs for the five most common *Candida* species isolates (3146 in total) from this study (n = 1705) and from three additional studies (two single-centre studies [12,13] and a multicentre study [14]). The Pfaller et al. study [13] included isolates selected to include wild-type and fluconazole and echinocandin-resistant strains, whereas the two other studies included unselected clinical isolates. The modal MIC is highlighted in underlined font. The consensus WT-UL is indicated by a dashed vertical line (Table 4).

Species	N	0.016	0.03	0.06	0.125	0.25	0.5	1	2	4	8	>8	nwt	%	Reference
*C. albicans*	896	5	91	574	213	10		2	1				3	0.3	This study
*C. albicans*	462	5	101	245	107	3	1						1	0.2	[12]
*C. albicans*	29		3	12	7	1	2	3	1				6	20.7	[13]
*C. albicans*	163	7	50	48	50	6	2						2	1.2	[14]
Aggregated excl. [14]	1387	10	195	831	327	14	3	5	2				10	0.7	
															
*C. glabrata*	475				5	319	148	2	1				3	0.6	This study
*C. glabrata*	120				3	54	59	3	1				4	3.3	[12]
*C. glabrata*	29					15	12	3	1				4	13.8	[13]
*C. glabrata*	60	2		1	10	22	16	5	1	2	1		9	15.0	[14]
Aggregated	684	2		1	18	410	235	13	4	2	1		20	2.9	
															
*C. krusei*	110					14	74	22					0	0.0	This study
*C. krusei*	24					2	10	10	2				2	8.3	[12]
*C. krusei*	19					3	121	4					0	0.0	[13]
*C. krusei*	29				1	2	10	16					0	0.0	[14]
Aggregated	182				1	21	215	52	2				2	1.1	
															
*C. parapsilosis*	78				1	23	44	2	1	6	1		8	10.3	This study
*C. parapsilosis*	249			1	22	127	92	7					0	0.0	[12]
*C. parapsilosis*	15				1	11	3						0	0.0	[13]
*C. parapsilosis*	108	6		1	3	10	38	24	15	6	5		26	24.1	[14]
Aggregated	450	6		2	27	171	177	33	16	12	6		34	7.6	
															
*C. tropicalis*	146				8	44	75	16		2	1		3	2.1	This study
*C. tropicalis*	73			1	3	36	24	8		1			1	1.4	[12]
*C. tropicalis*	21		1		3	10	3	2	2				0	0.0	[13]
*C. tropicalis*	40			2	3	9	12	8	5			1	1	2.5	[14]
Aggregated	280		1	3	17	99	114	34	7	3	1	1	5	1.8	
															
*C. dubliniensis*	117			15	64	33	4		1				1	0.9	This study

nwt: non-wild-type.

**Table 4:** Statistical and visual wild-type upper limit values (WT-UL, mg/L) for the five most common *Candida* spp. determined using the aggregated distributions presented in Table 3, single-centre data for *C. dubliniensis* and 97.5–99.9% of the modelled MIC distributions.

	Statistical WT-UL Values				Visual WT-UL	Consensus WT-UL
	97.5%	99.0%	99.5%	99.9%		
*C. albicans*	0.125	0.125	0.25	0.25	0.25	0.25
*C. glabrata*	0.5	0.5	0.5	1	1	0.5
*C. krusei*	1	1	1	1	1	1
*C. parapsilosis*	1	1	1	2	2	1
*C. tropicalis*	1	2	2	2	2	2
*C. dubliniensis*	0.25	0.5	0.5	0.5	0.5	0.5

**Table 5:** MIC and Fks protein details for isolates with elevated MICs to either ibrexafungerp or echinocandin.

Species	MIC (mg/L) *						Fks Amino Acid Alterations
	IBX	ANF	MFG	AMB	FLZ	VRZ	
*C. albicans*	2	0.008	0.03	0.125	0.125	0.004	WT
*C. albicans*	1	0.06	0.25	0.25	0.5	0.016	F641S
*C. albicans*	1	0.125	0.5	0.25	0.125	0.004	F641S
*C. albicans*	0.25	0.25	1	0.125	0.125	0.008	R1361H
*C. albicans*	0.25	0.008	0.03	0.25	0.25	0.008	
*C. albicans*	0.25	0.008	0.03	0.25	0.125	≤0.004	
*C. albicans*	0.25	0.004	0.016	0.25	0.25	0.008	
*C. albicans*	0.25	0.008	0.016	0.25	0.5	0.008	
*C. albicans*	0.25	0.25	2	0.25	0.25	0.008	S645P
*C. albicans*	0.25	0.004	0.016	0.25	0.25	0.008	
*C. albicans*	0.25	0.008	0.03	0.5	0.25	0.008	
*C. albicans*	0.25	0.008	0.016	0.125	0.125	≤0.004	
*C. albicans*	0.25	≤0.004	0.016	0.5	0.5	0.008	
*C. albicans*	0.125	0.03	0.06	0.25	0.25	0.008	R1361H
*C. albicans*	0.06	0.06	0.125	0.25	0.125	≤0.004	R1361S
*C. albicans*	0.06	0.125	0.25	0.25	0.25	≤0.004	R1361S
*C. albicans*	0.06	0.016	0.06	0.25	0.125	0.004	S645P
*C. dubliniensis*	2	0.016	0.03	0.06	0.5	0.016	WT
*C. dubliniensis*	0.5	0.016	0.03	0.06	0.5	0.016	
*C. dubliniensis*	0.5	0.03	0.06	0.25	0.5	0.016	
*C. dubliniensis*	0.5	0.016	0.03	0.25	0.5	0.016	
*C. dubliniensis*	0.5	0.008	0.016	0.06	0.25	0.016	
*C. glabrata*	2	0.5	0.5	0.25	2	0.03	F659del (Fks2)
*C. glabrata*	1	1	2	0.25	32	0.5	F659del (Fks2)
*C. glabrata*	1	0.03	0.06	0.25	2	0.06	F659L (Fks2)
*C. glabrata*	0.5	0.06	0.06	0.25	16	0.5	S629P (Fks1)
*C. glabrata*	0.5	0.06	0.06	0.125	4	0.06	L662F (Fks2)
*C. glabrata*	0.5	0.125	0.06	0.25	8	0.125	F659Y (Fks2)
*C. glabrata*	0.5	0.125	0.125	0.25	32	1	P667I (Fks2)
*C. glabrata*	0.5	1	1	0.25	4	0.125	S663P (Fks2)
*C. glabrata*	0.5	1	1	0.5	>64	4	S663P (Fks2)
*C. glabrata*	0.5	0.125	0.125	0.5	64	2	L662W (Fks2)
*C. glabrata*	0.5	2	2	0.25	4	0.06	S629P (Fks1)/L640X (Fks2)
*C. glabrata*	0.25	0.06	0.25	0.25	4	0.125	R665G (Fks2)
*C. glabrata*	0.25	0.125	0.06	0.25	2	0.06	F659Y (Fks2)
*C. glabrata*	0.25	0.03	0.06	0.25	2	0.06	K1323E 17 AA prior to Fks1 HS-2
*C. krusei*	1	0.125	2	0.5	32	0.5	S659P/L701M
*C. parapsilosis*	8	2	4	1	>16	>4	WT
*C. parapsilosis*	4	1	2	1	64	1	
*C. parapsilosis*	4	2	2	0.5	64	2	
*C. parapsilosis*	4	0.5	1	0.5	1	0.016	
*C. parapsilosis*	4	1	2	0.5	0.5	0.008	
*C. parapsilosis*	4	0.5	2	0.5	0.25	0.008	
*C. parapsilosis*	4	0.5	1	0.5	0.25	0.016	
*C. parapsilosis*	2	2	4	1	1	0.016	WT
*C. tropicalis*	8	0.016	0.03	0.5	0.5	0.03	WT
*C. tropicalis*	4	0.016	0.03	0.5	2	0.125	WT
*C. tropicalis*	4	0.016	0.016	0.125	0.5	0.03	WT
*C. tropicalis*	1	0.03	0.06	0.5	4	0.125	WT
*C. auris*	1	2	>4	1	>64	1	F635Y

* MICs that are categorised as non-wild-type for ibrexafungerp with all four WT-UL values and for the echinocandins adopting the EUCAST ECOFFs are indicated in red. Blue is used for Fks alterations at the first codon in hot spot one of the target genes, which has been associated with ibrexafungerp cross-resistance. Empty fields indicate that *FKS* sequencing has not been done.

**Table 6:** Susceptibility characteristics given as MIC_50_ and MIC range (both mg/L) and percentage of isolates classified as I (susceptible, increased exposure) ^a^ and R (resistant) for 1965 Danish yeast isolates collected 2020–2021 to five licensed antifungal agents.

	Anidulafungin				Micafungin				Amphotericin B				Fluconazole					Voriconazole		
Species (*N*)	MIC_50_	MIC Range	%R/nwt		MIC_50_	MIC Range	%R/nwt		MIC_50_	MIC Range	%R/nwt		MIC_50_	MIC Range	%I	%R		MIC_50_	MIC Range	%R/nwt
*Candida* spp.																				
*C. albicans* (896)	≤0.004	≤0.004–0.5	1.0		0.016	≤0.004–2	1.8		0.25	0.06–1	0		0.25	≤0.06–>64	0.3	2.7		0.008	≤0.004–>4	3.0
*C. dubliniensis* (117)	0.016	≤0.004–0.03	0		0.03	0.008–0.06	0		0.06	0.016–0.25	0		0.25	0.125–>64	0.0	2.6		0.008	≤0.004–>4	0.9
*C. glabrata* (475)	0.016	0.008–2	2.3		0.03	≤0.004–2	3.6		0.25	0.03–1	0		4	1–>64	89.9	10.1		0.06	0.016–>4	6.7
*C. krusei* (110)	0.03	0.016–0.125	0.9		0.125	0.06–2	0.9		0.5	0.125–1	0		32	16–>64	0	100.0		0.5	0.125–>4	4.5
*C. parapsilosis* (78)	1	0.25–2	0		1	0.5–4	2.6		0.5	0.125–1	0		1	0.25–64	2.6	6.4		0.016	0.008–>4	6.4
*C. tropicalis* (146)	0.016	≤0.004–0.03	0		0.03	0.008–6	0		0.5	0.125–1	0		0.5	0.125–32	5.5	2.7		0.03	0.008–1	5.5
*C. guilliermondii* (12)	0.5	0.25–1	NA ^b^		0.25	0.25–0.5	NA		0.125	0.125–0.25	0		8	2–>64	18.2	72.7		0.25	0.06–4	NA
*C. kefyr* (8)	0.03	0.016–0.125	NA		0.06	0.06–0.25	NA		0.5	0.25–1	0		0.5	0.25–1	0.0	0.0		0.008	0.008–0.03	NA
*C. lusitaniae* (19)	0.03	0.016–0.06	NA		0.06	0.06–0.125	NA		0.125	0.06–0.25	0		0.5	0.125–1	0.0	0.0		0.008	≤0.004–0.016	NA
Other spp. (34)	0.125	0.008–>4	NA		0.125	0.03–>4	NA		0.5	0.016–1	0		4	0.125–>64	11.8	44.1		0.125	≤0.004–>4	NA
Other yeast																				
*S. cerevisiae* (30)	0.06	0.03–0.125	NA		0.25	0.125–0.25	NA		0.25	0.06–0.5	0		8	2–32	20.0	76.7		0.125	0.06–1	NA
*Cryptococcus* spp. (7)	>4	>4	NA		>4	>4	NA		0.5	0.25–1	0		4	2–16	NA	NA		0.125	0.03–0.25	0
Rare yeast (34)	>4	0.008–>4	NA		>4	0.03–>4	NA		0.5	0.125–>4	11.8		16	0.25–>64	NA	NA		0.25	≤0.004–>4	NA

^a^ No isolates are classified as I to anidulafungin, micafungin, amphotericin B or voriconazole; hence, columns for the I-category are omitted for these agents. ^b^ NA: not applicable (EUCAST breakpoints or ECOFFs are not established). nwt: non-wild-type.

## Data Availability

Not applicable.

## Supplementary Materials

The following supporting information can be downloaded at: https://www.mdpi.com/article/10.3390/jof8101106/s1, Figure S1: Growth curve examples for isolates defined as ibrexafungerp wild-type and non-wild-type adopting the WT-UL 97.5%; Table S1: Ibrexafungerp EUCAST MICs of rare yeast.
