# Supplementary material for: EUCAST Ibrexafungerp MICs and Wild-Type Upper Limits for Contemporary Danish Yeast Isolates
Source: J Fungi (Basel). 2022 Oct 20;8(10):1106. doi: 10.3390/jof8101106 (PMC9605171; doi:10.3390/jof8101106)
Supplement: Supplementary file 1 [file jof-08-01106-s001.zip › jof-1931474-supplementary/Arendrup Ibrexa Suppl. Fig S1 Aberrant and classical growth curves Final for JoF 17-10-2022.pdf]

Figure S1. Growth curve examples for isolates defined as ibrexafungerp wild-type and non-wild-type adopting the WT-UL 97.5%

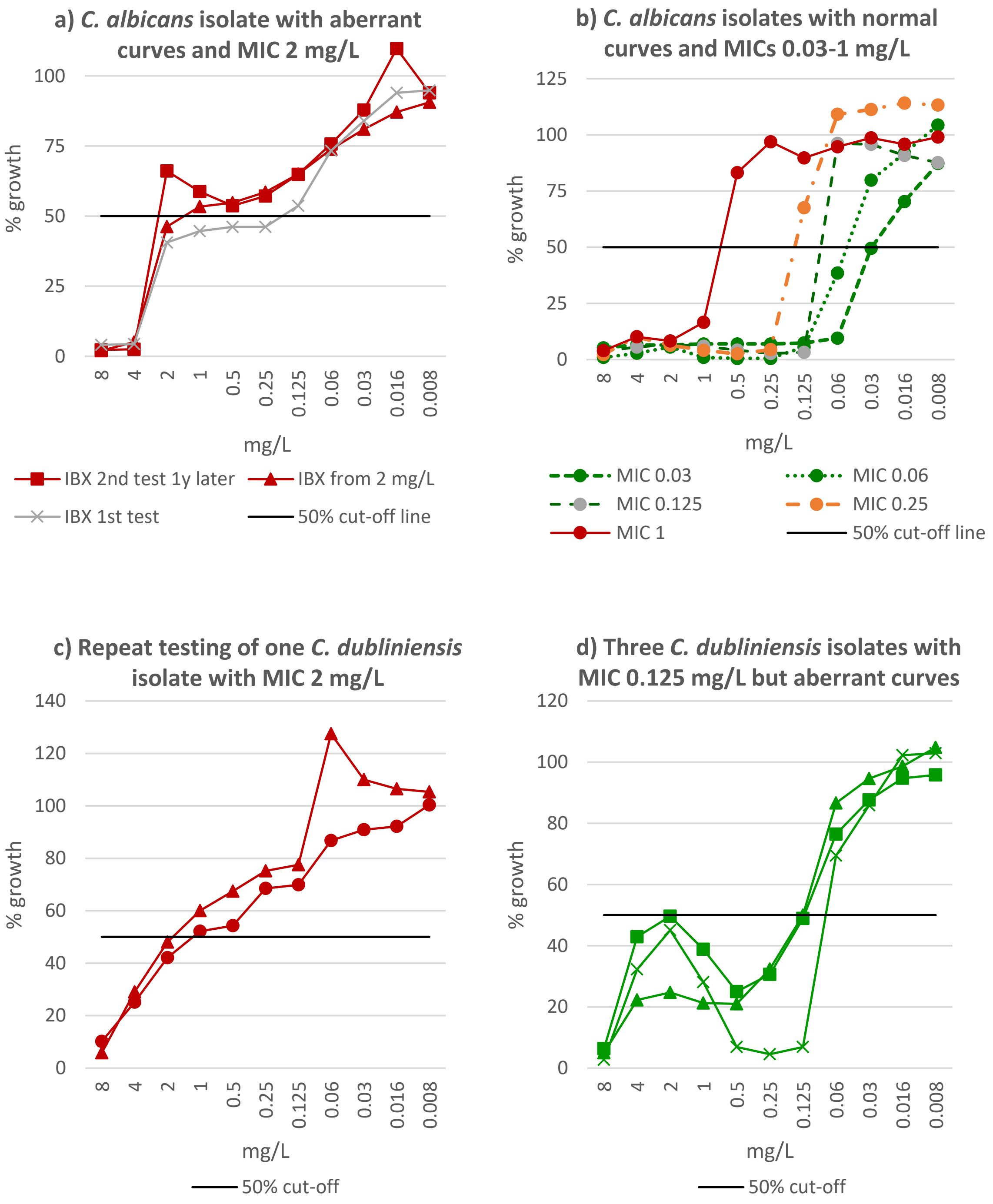

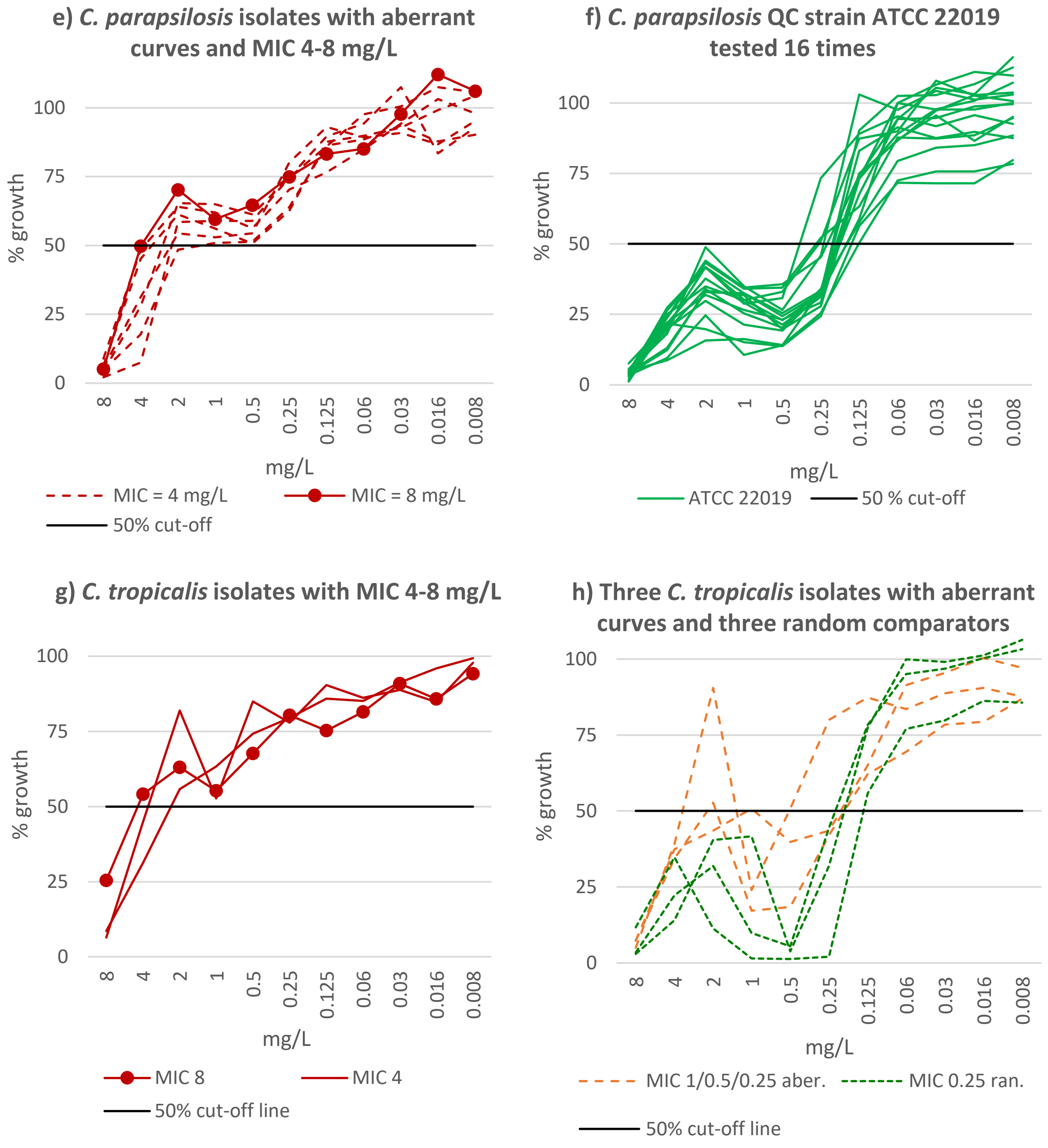

e) C. parapsilosis isolates with aberrant curves and MIC 4-8 mg/L
% growth
0
25
50
75
100
8
4
2
1
0.5
0.25
0.125
0.06
0.03
0.016
0.008
mg/L
MIC = 4 mg/L
MIC = 8 mg/L
50% cut-off
f) C. parapsilosis QC strain ATCC 22019 tested 16 times
% growth
mg/L
ATCC 22019
50 % cut-off
g) C. tropicalis isolates with MIC 4-8 mg/L
% growth
mg/L
MIC 8
MIC 4
50% cut-off line
h) Three C. tropicalis isolates with aberrant curves and three random comparators
% growth
mg/L
MIC 1/0.5/0.25 aber.
MIC 0.25 ran.
50% cut-off line
