# Supplementary material for: EUCAST Ibrexafungerp MICs and Wild-Type Upper Limits for Contemporary Danish Yeast Isolates
Source: J Fungi (Basel). 2022 Oct 20;8(10):1106. doi: 10.3390/jof8101106 (PMC9605171; doi:10.3390/jof8101106)
Supplement: Supplementary file 1 [file jof-08-01106-s001.zip › jof-1931474-supplementary/jof-1931474-supplementary.pdf]

Table S1. Ibrexafungerp EUCAST MICs of rare yeast.

		Ibrexafungerp MICs (mg/L)									
	N	**0.03**	**0.06**	**0.125**	**0.25**	**0.5**	**1**	**2**	**4**	**8**	**>8**
Candida species											
C. metapsilosis	1			1							
C. orthopsilosis	8			1	6	1					
C. kefyr	8			1	5	2					
C. lambica	1				1						
C. norvegensis	6				6						
C. pelliculosa	2				2						
C. utilis	1				1						
C. guilliermondii	11					1	9	1			
C. intermedia	1					1					
C. stellimalicola	1					1					
C. auris	2					1	1				
C. fermentati	4						4				
C. inconspicua	1						1				
C. lipolytica	1						1				
C. melibiosica	1						1				
C. pulcherrima	1						1				
C. sorbosivorans	1						1				
C. lusitaniae	19						7	11	1		
C. pararugosa	1									1	
Other yeast											
Arxula adeninivorans	1	1									
Saccharomyces cerevisiae	30			1	10	19					
Lodderomyces elongisporus	1				1						
Wickerhamomyces anomalus	2				2						
Pichia manshurica	1					1					
Wickerhamomyces onychis	1					1					
Yarrowia lipolytica	1					1					
Kodamaea ohmeri	1						1				
Magnusiomyces capitatus	5						2	1		2	
Wickerhamomyces species	1						1				
Cryptococcus neoformans spp. complex*	7							5	2		
Microsporum audouinii	3							2	1		
Trichosporon asahii	4							1	1	2	
Geotrichum candidum	5								1	2	2
Rhodotorula mucilaginosa	3								1	1	1
Exophiala dermatitidis	1									1	
Lachancea thermotolerans	1									1	
Trichosporon dermatis	2									2	
Geotrichum capitatum	2									1	1
Geotrichum species	2									1	1
Geotrichum silvicola	1										1

*Six *Cryptococcus neoformans* and one *Cryptococcus neoformans* spp. complex.
